# Allometric scaling of skin thickness, elasticity, viscoelasticity to mass for micro-medical device translation: from mice, rats, rabbits, pigs to humans

**DOI:** 10.1038/s41598-017-15830-7

**Published:** 2017-11-21

**Authors:** Jonathan C. J. Wei, Grant A. Edwards, Darren J. Martin, Han Huang, Michael L. Crichton, Mark A. F. Kendall

**Affiliations:** 1 Delivery of Drugs and Genes Group (D2G2), Australian Institute for Bioengineering and Nanotechnology, The University of Queensland, St Lucia QLD, 4072 Australia; 2 Martin group, Australian Institute for Bioengineering and Nanotechnology, The University of Queensland, St Lucia QLD, 4072 Australia; 3 Nanomechanics and Nanomanufacturing Group, School of Mechanical and Mining Engineering, Faculty of Engineering, Architecture and Information Technology, The University of Queensland, St Lucia QLD, 4072 Australia; 4 Institute of Mechanical, Process and Energy Engineering, School of Engineering and Physical Sciences, Heriot-Watt University, Edinburgh, EH14 4AS United Kingdom; 5 ARC Centre of Excellence in Convergent Bio-Nano Science and Technology, The University of Queensland, St Lucia QLD, 4072 Australia; 6 Faculty of Medicine, The University of Queensland, Royal Brisbane and Women’s Hospital, Herston QLD, 4006 Australia

## Abstract

Emerging micro-scale medical devices are showing promise, whether in delivering drugs or extracting diagnostic biomarkers from skin. In progressing these devices through animal models towards clinical products, understanding the mechanical properties and skin tissue structure with which they interact will be important. Here, through measurement and analytical modelling, we advanced knowledge of these properties for commonly used laboratory animals and humans (~30 g to ~150 kg). We hypothesised that skin’s stiffness is a function of the thickness of its layers through allometric scaling, which could be estimated from knowing a species’ body mass. Results suggest that skin layer thicknesses are proportional to body mass with similar composition ratios, inter- and intra-species. Experimental trends showed elastic moduli increased with body mass, except for human skin. To interpret the relationship between species, we developed a simple analytical model for the bulk elastic moduli of skin, which correlated well with experimental data. Our model suggest that layer thicknesses may be a key driver of structural stiffness, as the skin layer constituents are physically and therefore mechanically similar between species. Our findings help advance the knowledge of mammalian skin mechanical properties, providing a route towards streamlined micro-device research and development onto clinical use.

## Introduction

With its highly accessible and abundant biological environment, the skin is an attractive site for a range of therapeutic applications for medical devices designed to improve healthcare. These include devices delivering drugs and vaccines into the skin^1,2^ and, alternatively, extracting skin biomarkers and electrical signals for diagnosis of disease^3^. In recent decades, the field has progressed beyond the needle and syringe towards more precise, minimally-invasive, micro-devices that exploit the mechanical properties of skin.

However, many existing and emerging micro-devices^4,5^ target the skin at scales and strain-rates distinct to the needle and syringe, exposing gaps in the knowledge of key mechanical properties. These knowledge gaps are further exacerbated as the path of medical device development progresses through typical animal models (e.g. mice, rats, rabbits and pigs) through to human clinical testing. To put simply, how do the key structural and mechanical properties of skin relate between different species? Identifying these properties would not only fill fundamental knowledge gaps, but could also streamline medical device research and development with reduced usage of animals and potentially smaller clinical trials.

Looking more closely at the challenge: it is well-established that skin is a complex, viscoelastic, biological composite structure, consisting of the epidermis (E), dermis (D) and hypodermis (H) or the subcutaneous tissue. The epidermis is further divided into stratum corneum (SC), a tough, physical and elastic barrier, comprised of keratinised dead skin cells, which makes accessing the layers beneath difficult^6^. This is made even more challenging by the strain-rate dependent, viscoelastic viable epidermis (VE) – the uppermost living layer of the skin and dermis, where collagen, blood capillaries, antigen presenting cells (APCs) and biomarkers are located, often important for drug delivery and diagnostics^7^. It is clear that the composition of skin is fundamental to its mechanical properties. However, because of strain-rate dependency, it is difficult to compare data directly with different experimentation protocols. Moreover, these skin layers differ in thickness between species, as shown in Table 1 (and a further table by Hirschberg *et al*.), where various measuring techniques and skin sites were used^8^.

**Table 1:** Selection of mean skin thicknesses of selected species reported in literature for non-weight bearing sites (mean ± SD/SE*).

Species	Site	SC		VE		D		Source
		µm	SD/SE*	µm	SD/SE*	µm	SD	
**Mouse**	Dorsum	9	—	29	—	662	—	48
**Mouse**	Buttock, ear, shoulder, back, abdomen (paraffin)	3.38	±0.30*	11.50	±1.24*	—	—	48
**Mouse**	Buttock, ear, shoulder, back, abdomen (frozen)	6.69	±0.96*	9.24	±0.96*	—	—	48
**Mouse**	Back	~5	—	~21–22	—	~275–280		73
**Rat**	Dorsum	18	—	32	—	2040	—	48
**Rat**	Buttock, ear, shoulder, back, abdomen (paraffin)	4.04	± 0.47*	15.34	± 1.21*	—	—	48
**Rat**	Buttock, ear, shoulder, back, abdomen (frozen)	9.91	±1.14*	10.70	±1.73*	—	—	74
**Rabbit**	Lumbar dorsum	11.7	±3.6	20.6	±4.0	2174.0	±486.7	74
**Rabbit**	Lumbar dorsum	9.5	±1.6	19.4	±4.8	1719.3	±258.5	74
**Rabbit**	Buttock, ear, shoulder, back, abdomen (paraffin)	6.89	±0.88*	13.83	±1.23*	—	—	48
**Rabbit**	Buttock, ear, shoulder, back, abdomen (frozen)	10.91	±1.48*	9.39	±1.25*	—	—	48
**Pig**	Buttock, ear, shoulder, back, abdomen (paraffin)	12.85	±1.19*	53.17	±3.19*	—	—	48
**Pig**	Buttock, ear, shoulder, back, abdomen (frozen)	41.33	±3.73*	15.37	±1.51*	—	—	48
**Pig**	Ear	17–28		60–85		1440–2210	(incl. H)	75
**Human**	Abdomen	17	—	47	—	2906	—	48
**Human**	—	10	—	50–120	—	2.28	—	76
**Human**	Various sites	—	—	31–637	(incl. SC)	521–1977	(E+D)	41

One particular example of emerging micro-devices susceptible to skin differences is the microneedle class of devices for drug/vaccine delivery and wearable diagnostics. The therapeutic attributes of many types of microneedles have been established in different animal models – including mice (e.g.^9,10^), rats (e.g.^11,12^), rabbits (e.g.^13,14^), and pigs (e.g.^15,16^). However, how these therapeutic attributes are translated to widespread human testing (e.g.^17,18^) is reliant upon understanding how the key skin mechanical properties change between the species. Indeed, so far, microneedle utility in humans is limited, with extrapolated designs empirically derived for humans with varying degrees of success^19^. For example, Li *et al*. reported difficulty in penetrating through human skin, compared with rat skin, using the same application conditions^12^. A reason for this may be the lack of data to aid design translation to humans. Another reason may be that skin thicknesses vary greatly between the size of species, e.g. the SC is ~5 µm in mice to ~10–20 µm in humans, and also between sites within the same species^20^. This is despite almost all species possessing the same distinct epidermal, dermal and hypodermal layers^21^.

A challenge in tissue mechanical property characterisation (such as elastic moduli) is that they often depend on test methods – orientation of loading conditions^22^, state of the sample (e.g. *in vivo*, *ex vivo*)^23^, testing rate^24^ and contact area^25^. As a result, for human skin, the reported elastic modulus can vary from 0.4–0.8 MPa for torsion tests, 4.6–20 MPa for tensile tests and 0.05–0.15 MPa for suction tests, according to Pailler-Mattei *et al*.^26^ Because of these factors, results from various skin studies are difficult to compare.

Nevertheless, within given species, the knowledge of relevant skin mechanical properties has been advanced – for example on elasticity^27,28^, fracture energy^29^ and percutaneous absorption^30^. However, in terms of mechanical properties of biological tissues, we are not aware of allometric investigations (the study of physiological characteristics relating to body size) of key mechanical properties of skin, ranging across body masses spanning orders of magnitudes (from ~30 g to ~150 kg), using a consistent approach. In this paper, we measured key skin physical and mechanical properties at relevant sites – thickness and elastic moduli at the microscale interface – using micro-indentation across five mammalian species of regular use in medical device research and development. Namely: mice, rats, rabbits, pigs (small and large) and humans (*in vivo* and *ex vivo*). Past indentation studies has shown the importance of accounting for tip-sample contact size for viscoelastic materials^31^ however, flat tips have been used for the benefit of analysis simplification^28^. Stress distribution on cartilage tissue was compared for flat and spherical tips with a cell death model^32^ and that while higher local stresses were experienced around the flat tip circumference, flat tips still provided comparable elastic moduli between the two tip profiles.

Our hypothesis is that skin of common laboratory animals – and humans – is mechanically a function of the layer thicknesses of each skin layer, and also related to body mass. To test this hypothesis, we first measured the SC, VE and dermis layer thicknesses. We then measured elasticity and viscoelasticity of full thickness skin. Finally, we aggregated these data to develop a simple model for estimating the elastic modulus of skin by knowing a basic parameter – the skin’s layer thicknesses (relating to body mass). This relationship could be applied to more streamlined testing of mechanical micro-devices and other related work spanning from small to large animal models and indeed humans.

## Results

To characterise the skin of different species, we first measured the thicknesses of the key skin layers – the SC, VE and dermis of mice, rats, rabbits, pigs and humans. We then indented the skin using a range of probes to measure the elastic moduli with consideration for viscoelastic effects, and used an analytical spring model to test the hypothesis that the elastic modulus is mainly dependent on skin thickness. We defined elasticity, modulus and material stiffness as the elastic modulus *E*, whereas the structural, axial or layer stiffness, dependent on the geometry of the material, *k*. The full skin thickness comprising the SC, VE and dermis was measured as a full thickness material interfacing medical devices, and analysed as a composite material with the three distinct layers^33^. Values reported herein are mean ± standard deviation.

### Morphology

#### Skin layers and thicknesses

**Figure 1:**
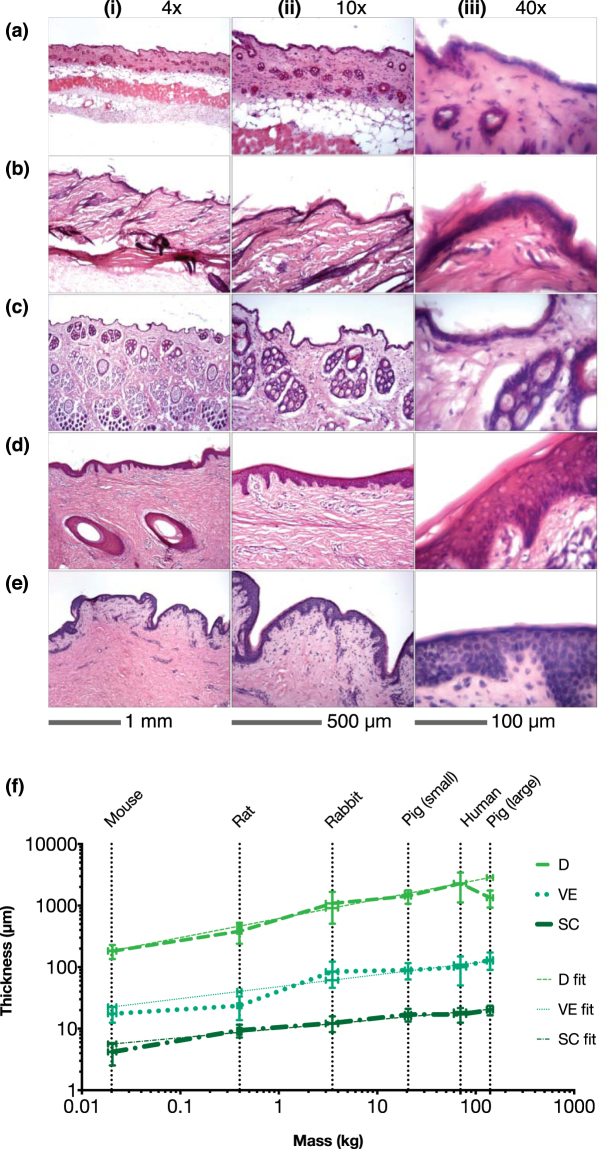
Representative cryo histological cross sections of upper skin specimens: (**a**) mouse – flank, (**b**) rat – flank, (**c)** rabbit – flank, (**d**) large pig – ear (**e**) human – abdomen, at three magnification levels (i) 4x, (ii) 10x and (iii) 40x. (**f**) Measured skin strata thicknesses based on histology plotted against species mass. Horizontal error bars show SD of mass and vertical error bars show SD of measured thicknesses.

Skin layer thicknesses measured using histology sectioning are shown in Fig. 1. Stained sections show structure, i.e. skin layers and cells residing within the skin strata, which were used as a guide to identify the skin layers. The SC is the top, stratified, corneocyte layer that is pink in colour visible in the 40x images Fig. 1(a–e iii **)**. The VE is the layer beneath the SC in dark purple, which appears undulating at the lower boundary. Individual cells and nuclei (dark purple) can also be seen in higher magnifications. The dermis is the light purple region below the VE, which is the thickest of the three. Hair follicles/shafts (circular/oval white spaces in the dermis) can also be identified in all species apart from human skin.

Thickness measurement are collated in Table 2 and shown on a logarithmic scale vs. mass (Supplementary Table S1) in Fig. 1(f). While the three skin layers possess comparable thickness ratios between species (mean of ~1:4:95 of SC:VE:D), different features are observed between the species, which include:

- Rabbit dermis contains large numbers of hair follicles at ~4–5 mm^−2^, in contrast to pig dermis at <1 mm^−2^.
- Hair follicle shafts of pig skin are larger than that of smaller species.
- Hair follicles were not observed on human skin sections.
- The rabbit skin features a relatively thick epidermal layer, closer to pigs than mice.
- Pigs of higher body mass (~130–150 kg) appear to have thicker VE than smaller pigs (~20 kg). The overall dermis thickness between both groups remain close (P = 0.6806).

**Table 2:** Measured skin layer thicknesses for comparison with literature values in presented Table 1.

Species	Site	SC		VE		D		Approximate total
		*t* (µm)	SD	*t* (µm)	SD	*t* (µm)	SD	*t* (µm)
Mouse	Flank	4.19	±1.79	17.50	±4.98	182.4	±46.72	204
Rat	Flank	9.38	±2.21	23.58	± 9.79	382.42	± 142.49	415
Rabbit	Flank	12.32	±3.52	84.34	±38.28	1085.85	±578.52	1183
Pig (small)	Ear	17.01	±3.96	89.60	±26.70	1423.94	±522.80	1531
Pig (large)	Ear	20.02	±3.55	131.50	±41.29	1340.59	±411.79	1492
Human	Abdomen	17.07	±4.56	99.80	±49.29	2284.05	±1161.64	2401

Calculated *P*-values of layer thickness comparisons are provided in Supplementary Table S2. Notably, mice and rat skin are alike at ~20 µm in the VE, as well as that of rabbits and small pigs despite the large differences in mass (~20 x and ~6 x respectively). From the measurements of skin layer thicknesses, allometric scaling relationships fitted to experimental trends are presented as power law curves in Table 3(i) for skin thickness interpolation, if the mass of an animal is known.

**Table 3:** Parameters for fitted power law curves ($y=a{x}^{b}$) of measured skin layer thicknesses $y$ (µm) with respect to species mass $x$ (kg).

Parameter	*a*	*b*	*R* ^*2*^
**(i) Skin layer (with large pigs)**			
Stratum corneum	10.01	0.143	0.96
Viable epidermis	47.7	0.202	0.91
Dermis	756	0.187	0.71
**(ii) Skin layer (without large pigs)**			
Stratum corneum	9.98	0.147	0.97
Viable epidermis	48.1	0.194	0.85
Dermis	617	0.310	0.98

### Skin mechanical properties

#### Viscoelastic properties

**Figure 2:**
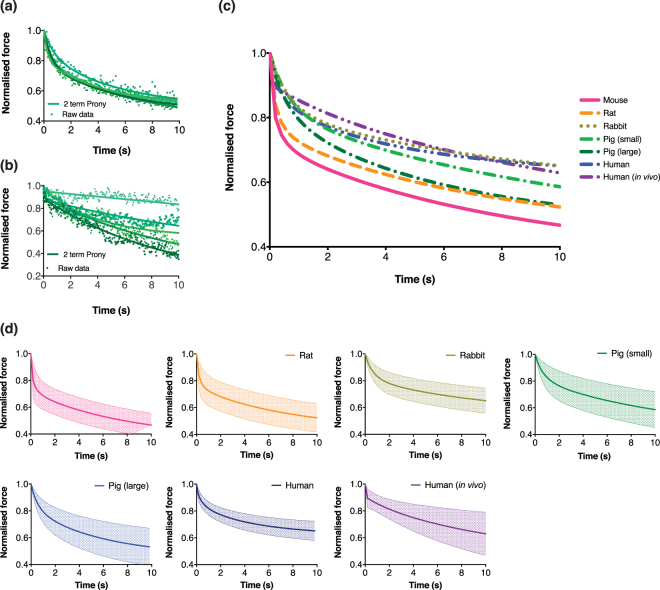
(**a**) Representative examples of two-term Prony series fitted to raw data showing the spread of raw data and curve fits. **(b**) Representative example of fitted two-term Prony series on one set of human *in vivo* data illustrating noticeable oscillations caused by heartbeats and small body movements of volunteers (first 10 s shown). Curves were still able to fit to the raw data as shown. (**c**) Mean force-time response of skin during a step-load over the first ten seconds of all species. (**d**) Mean and SD of force-relaxation curves for each species shown individually.

Viscoelastic properties provide information on skin deformability at different strain rates and were required to obtain the elastic properties independent of the indentation rate. Figure 2(a) shows examples of the typical fit of Prony series to a subset group of raw data (human skin, indentation rate *v* = 0.1 mm s^−1^, tip radius *R* = 0.18 mm). Prony series goodness of fit (*R* ^2^) are presented in Supplementary Table S3. Higher noise and spread can be seen in human skin experiments due to the lower than expected load measured (hence higher relative noise from the load cell and lower *R* ^2^). Small body movements and breathing from volunteers from *in vivo* human skin group were detectable and an example is shown in Fig. 2(b). Because of this, it was more difficult to fit curves to the human *in vivo* tests and more optimisation had to be performed by adjusting the initial fitting parameters for the results to converge, which could suggest that the relaxation profiles are noticeably different to the rest of the species in Fig. 2(b). Raw force-time data of every replicate for each species is plotted in Supplementary Fig. S1.

Immediately after a step load application, we observed a transient, rapid drop of resistance force against the tip over the first second for all skin, followed by a steady-state, plateauing decrease for the remainder of the experiment. The first ten seconds (transient and some steady state phases) that are most applicable to skin-targeting devices are shown in Fig. 2(c,d). This behaviour was observed throughout the experiments for all skin types. The figure shows the force decreasing over ten seconds immediately after a 100 µm s^−1^ step load when the tip is held at constant (maximum) displacement of approximately 10% of the skin thickness. We observed that the skin of smaller species (e.g. mice, rats) show more viscoelastic effects than larger species (e.g. humans), i.e. the force decreases more over the same period (mean residual force after ten seconds is ~40% for mouse skin but ~80% for *in vivo* human skin). The *in vivo* human skin appeared not to relax as much compared to the other groups initially, but the at ten seconds it appears to continue decreasing more than the *ex vivo* human skin. Comparing between small and large pigs, the larger showed higher relaxation compared with the smaller. The Prony series parameters *τ*_1_, *τ*_2_, *g*_1_, *g*_2_ are produced in Table 4 and their resulting *g(t)* coefficient at 100 mm s^−1^ are given in Supplementary Table S4 for reference.

**Table 4:** Prony series parameters *τ*_*1-2*_, *g*_*1-2*_ (mean ± SD).

Coefficient ± SD	*g*_*1*_	*g*_*2*_	*τ*_*1*_	*τ*_*2*_
Mouse	0.333 ± 0.73	0.355 ± 0.08	0.227 ± 0.19	8.394 ± 3.09
Rat	0.338 ± 0.50	0.307 ± 0.07	0.156 ± 0.07	8.206 ± 13.37
Rabbit	0.246 ± 0.06	0.263 ± 0.05	1.770 ± 3.28	8.457 ± 3.97
Pig (small)	0.378 ± 0.08	0.419 ± 0.05	0.853 ± 0.14	7.625 ± 3.81
Pig (large)	0.219 ± 0.06	0.376 ± 0.14	0.515 ± 0.15	6.496 ± 7.28
Human	0.142 ± 0.04	0.236 ± 0.08	0.423 ± 0.27	7.198 ± 5.55
Human *in vivo*	0.100 ± 0.07	0.713 ± 0.43	0.034 ± 0.12	12.053 ± 7.44

#### Elastic properties

**Figure 3:**
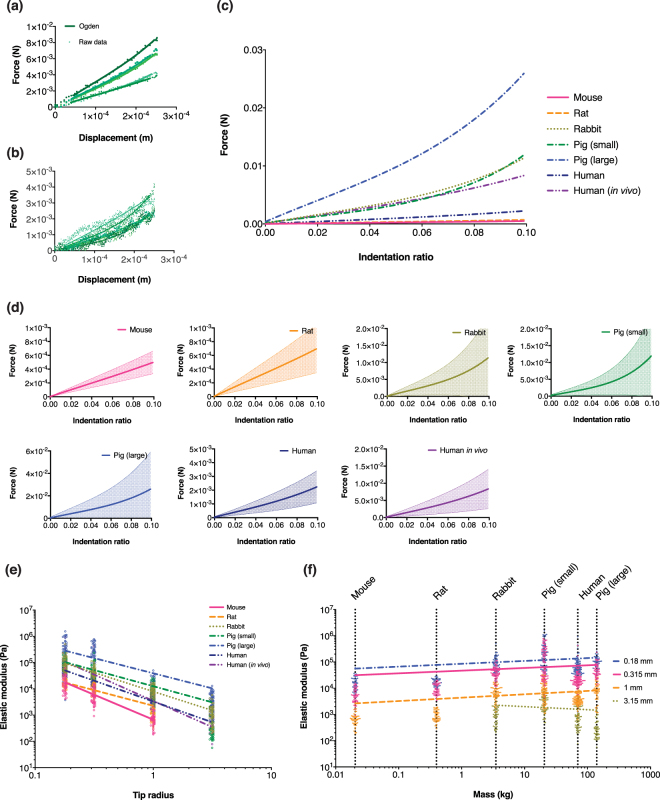
(**a**) Representative examples of raw data fitted to Ogden model. (**b**) Representative example of force-displacement curves when measuring human *in vivo* illustrating cyclical vibration caused by volunteers. (**c**) Mean force-displacement curves from all indents. (**d**) Mean force-displacement fitted to Ogden model normalised to 10% strain for all species with SD in shaded region. Note that y-axes have different scales. (**e**) Elastic moduli experimental trends shown vs. indentation tip radii with individual data points. (**f**) Elastic moduli shown vs. body mass.

The elastic moduli of skin were calculated as per Materials and methods - indentation procedure, accounting for the viscoelastic effects quantified previously. Figure 3(a) shows a typical fit of the Ogden model to raw data and Fig. 3(b) shows an *in vivo* human skin example. Greater data spread and noise were captured during the indentation because of volunteer movements. Oscillations were likely caused by breathing, as observed that each inhalation corresponded to a slight rise of the load (and vice versa with exhalation and slight drop), increasing the difficulty in the Ogden fit. Despite this, both Prony and Ogden curves were still able to be fitted to the mean of the sinusoidal heart beat to obtain mechanical properties. Summaries of the goodness of fit and the *α* coefficients are shown in Supplementary Tables S5 and S6 respectively. Raw force-displacement curves of every replicate for each species are shown in Supplementary Fig. S2.

Figure 3(c) shows mean and standard deviation force-displacement curves for all indents of all species. Curves followed behaviour typical of hyperelastic materials – linearly increasing for initial small displacements, then increasing more rapidly at large strains. Curves did not show a sudden drop in force during loading, which would have suggested puncturing of the skin. While all skin types followed the same loading path for the initial 0–5 µm indent, the human skin curves (both *in vivo* and *ex vivo*) had lower modulus compared with the other species. The maximum load at ~10% strain (maximum displacement) for human skin *in vivo* is 8.3 ± 5.7 mN and 2.24 ± 1.17 mN for human skin *ex vivo*, compared to 25.9 ± 33.3 mN for large pigs, 11.9 ± 12.2 mN for small pigs, 11.3 ± 12.5 mN for rabbit, 0.694 ± 0.348 mN for rat and 0.497 ± 0.164 mN for mouse.

In Fig. 3(e), we observed elastic modulus decreased with increasing tip radius in a log-log relationship. These trends were fitted as power curves in Table 5(i). Elastic modulus also increased with respect to species size for a given tip contact area, except for human skin being lower than rabbit skin. Large pig skin has the highest modulus of all tested species, whereas the skin’s modulus of small pigs followed more closely to other species. Such spread and variation of the elastic moduli were also reported in the indentation work of Ranamukhaarachichi *et al*.^33^, for example. Supplementary Fig. S5 shows the individual pig skin elastic moduli that pigs 4–5 had elastic moduli approximately one order of magnitude higher than pigs 1–3.

**Table 5:** Parameters for fitted power law curves ($y=a{x}^{b}$) of elastic moduli $y$ (Pa) **(i)** measured and **(ii)** analytically modelled with respect to body mass $x$ (kg) or (iii) tip radii $x$ (mm).

Parameter	*a*	*b*
**(i) Body mass for various tip radii (measured)**		
0.18	85704	0.1097
0.315	46881	0.1023
1	4375.2	0.1288
3.15	1380.4	−0.1179
**(ii) Body mass for various tip radii (analytical model)**		
0.001	8.2985 × 10^6^	0.0221
0.01	1.7701 × 10^6^	0.0284
0.1	3.0903 × 10^5^	0.0342
0.18	1.9275 × 10^5^	0.0359
0.315	1.2134 × 10^5^	0.0359
1	45920	0.0359
3.15	17061	0.0359
**(iii) Tip radius for various species (measured)**		
Mouse	672.98	−1.900
Rat	2275.1	−1.186
Rabbit	7943.3	−1.441
Pig (small)	12618	−1.227
Pig (large)	39355	−1.152
Human	3311.3	−1.584
Human (*in vivo*)	3499.5	−2.002

Table 6 shows that *ex vivo* human skin group had very low elastic moduli compared to other species in Fig. 3(c). To verify these were not anomalous samples, two additional *ex vivo* samples were repeated. A further group of *in vivo* human skin also was tested. *In vivo* human skin did not follow typical viscoelastic behaviour, i.e. slower tests gave higher measured forces, despite most other cases showed higher modulus with the faster indentation rate. The allometric scaling relationship to body mass is shown in Fig. 3(f), with curve fits presented in Table 5(iii).

**Table 6:** Summary of mean elastic moduli for all species.

Tip radius (mm)	0.18		0.315		1		3.15	
Species	*E* (kPa)	SD	*E* (kPa)	SD	*E* (kPa)	SD	*E* (kPa)	SD
Mouse	13.22	6.8	3.97	1.6	0.59	0.2		
Rat	17.04	5.2	9.98	4.6	0.97	0.5		
Rabbit	94.12	74.0	42.17	21.7	7.79	4.1	1.29	1.1
Pig (small)	102.52	47.2	55.09	38.9	9.70	6.4	0.43	0.4
Pig (large)	274.68	309.4	174.69	201.9	10.55	9.0	3.68	2.6
Human	50.26	19.3	19.83	7.1	4.74	1.9	1.99	1.1
Human (*in vivo*)	108.19	140.3	35.42	60.6	3.07	1.5	0.58	0.3

### Linear analytical model

To further help interpret the experimental trends, a simple model was used to help express the skin as three springs in series, isolating the elastic component. Skin properties of mice from Crichton *et al*.^33^ were used for each skin layer to estimate the elastic moduli and displacement of each animal and human model to test our hypothesis that the main influence of skin elasticity is the skin layer thicknesses.

**Figure 4:**
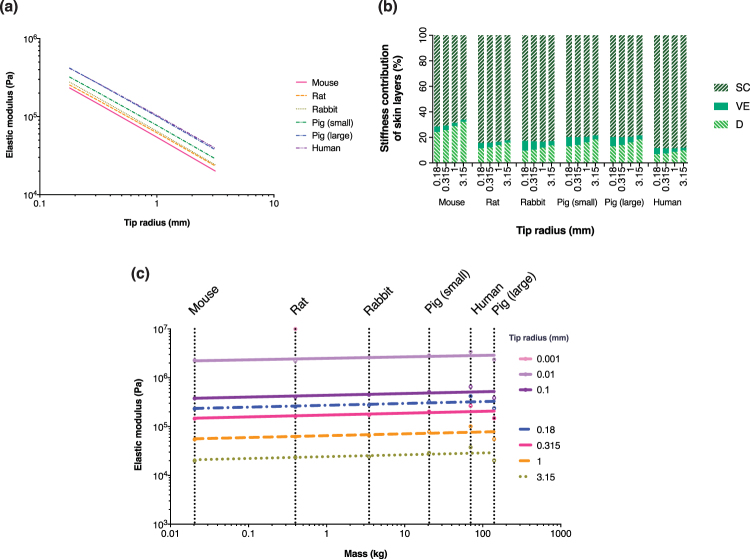
(**a**) Analytical model estimation of the elastic moduli against tip radii. (**b**) Approximate share of structural modulus of SC, VE and dermis for each species and tip size estimated from the analytical model using Equation 14. (**c**) Elastic moduli shows vs. body mass, for tip sizes used experimentally in this study and also estimates down to the cellular scale.

Figure 4(a) shows spring model elastic moduli was in similar orders of magnitude compared with Ogden fitted experimental data, matching more closely towards smaller tip contact areas. This established a relationship where the species mass (which leads to skin thickness) could be used to determine the skin’s elastic modulus from experimental trends. Figure 4(b) shows that the amount of load taken up by the SC was approximately 65–90%, followed by the VE at 5–25% and the VE at 1–8%, where the modulus contribution to the full thickness skin can be seen. Increasing the tip radius reduced the modulus contribution by the dermis, but was the opposite for the epidermal skin layers. Finally, the analytical model also showed that the dermis of larger species made less contribution to the overall modulus. For comparison Fig. 4(c) shows the analytical allmometric scaling estimates for elastic modulus by body mass.

## Discussion

The main purpose of this paper is to identify a way in which skin-interacting micro-devices can be translated easily between animal species to humans, and in the process, report on the physical, elastic and viscoelastic properties of skin across species. We hypothesised a relationship between species, so that their skin’s thickness and mechanical properties could be estimated simply by knowing the body mass of that species through interpreting the data using a simplified analytical model.

Our results established – for the first time – experimentally-determined trends between these properties across four commonly used laboratory animal models to humans, spanning from micrometres to millimetres on skin thickness and body mass ranging from ~30 g to ~150 kg. We are not aware of such a broad study of this sort in present literature, on key properties with an allometry theme. Indeed, Wang *et al*.^34^, reported structural and mechanical properties (thickness, stiffness and modulus over body weight and skin sites) of only one species (mouse model) under compression. The concept of allometry was introduced over a century ago, and more specifically, allometric scaling models for organisms’ body mass and metabolic rates were proposed in recent decades. This concept was further discussed on the applicability of *in vitro* models by Ahluwalia^35^. We propose to extend this relationship further to other physiological parameters in this study, namely, correlating skin’s thickness and its mechanical properties to body mass.

In terms of variation in the spread of data observed (for example, in Fig. 3(d)) are commonly encountered with biological specimens^36,37^, when compared to standard engineering materials (e.g. steel, rubber). However, substantial replicate variation, heartrate and respiration were also apparent in the indentation works of Miller *et al*.^45^ and Sridharan *et al*.^38,39^, consistent with our results. Ranamukhaarachchi *et al*. also reported higher scatter in elastic modulus during indentation tests in porcine vs. human skin^27^. We propose this could be due to environmental factors and skin layer thickness ratios. To boost data confidence, five specimens with five repeats per condition (indentation tip size and speed), with an additional two specimens for human *ex vivo*, were incorporated into the experimental design. Indeed, scatter of soft tissue data were also commented by Mattei and Ahluwalia^40^, who discussed potential sources for variation that could affect measured mechanical properties. They also presented guidelines on minimising this effect, such as using animals from controlled environments (e.g. same breeding facilities), using same test methods and minimising sample preservation period were implemented in this study ensuring reproducible data that is applicable and comparable across the scientific community.

In terms of the indentation method and the pre-load using the Instron, except for human skin, the pre-load (~1 mN) was much smaller (~20x) the peak loads experienced during indentation. The pre-load was the smallest possible load used as a reference point to commence indentation and was unlikely to result in any significant changes to the data. While the pre-load was relatively large for the case of human skin, the properties of the skin was still measured and that the elastic modulus was the lowest of all skins tested using the Instron. A significant pre-load magnitude would however likely result in an increase in mechanical properties^29^, preventing comparisons with other skins.

On the relation between skin thickness and body mass, we observed a proportional trend, as shown in Fig. 1(f). An exception is the dermis of the larger pigs is thinner than the smaller pigs, although the overall skin thickness was observed to be relatively close to each other. One explanation is that skin thickens up to a certain age or mass and because these pigs were raised for consumption (hence significantly increased body mass), their mass deviated away from their “normal” body mass over a relatively short period of time. For an experimental correlation of skin layer thicknesses relative to body mass, if we modify Table 3(i) to exclude larger pigs, we obtain a better-fitting allometric relationship (Table 3(ii)) plotted in Fig. 1(f).

Despite proposing this trend, it may not work well with species faraway from the boundaries of our chosen species, which could be a subject of further investigation. Nevertheless, all five species selected exhibited comparable ratios of skin layer thicknesses, i.e. mean of ~1:4:95 of SC:VE:D. This agrees with Lee and Hwang’s reported ratios (3.7–16.8% of epidermis within the entire human skin for most regions)^41^.

In terms of factors affecting skin thickness: previous studies have shown that skin site, age^42^; gender^20^, BMI^43^ and SC hydration^44^ as possibilities, however, we propose that these differences are likely to be smaller than comparing across species (except weight-bearing extremities e.g. thicker SC around the palms and soles than the arms and abdomen)^14^. For instance, full-thickness human skin can decrease in thickness by ~300 µm between 20 to 90 years of age^45^, which is only up to ~10% difference of its full thickness. In this study, human skin thickness data exhibited the highest coefficient of variation of up to 50%; the highest among other species. This high inter-donor dependency was also observed in Mattei *et al*.’s study on liver tissue structure^46^, who also commented that this was typical of human derived-samples. This could be minimised by using animals from the same sources, as with this paper (apart from human skin). While we compared different sites of animals, low intra-donor dependency was expected. This is supported by Lee *et al*. who showed that skin of the abdomen region is similar in thickness to the forearm (1332 ± 254 µm, vs. 1133 ± 215 µm respectively) and most other parts of the body^41^. We confirmed this in pigs to ensure this was the case with three sites on pig skin (data shown in Supplementary Fig. S6). The closest body of work we are aware of comparing tissues cross-species was by Malda *et al*. investigating the relationship between articular cartilage thickness and body mass^47^ and Monteiro-Riviere comparing the epidermal thicknesses of various species^48^.

On viscoelasticity, the skins of the smaller animals (i.e. mice, rats) were found to be more viscoelastic than those of larger species (Fig. 2(c)), which we hypothesised this was likely due to considerably higher relative thickness of the VE *vs*. dermis in smaller species (Table 2). Our relaxation profiles *cf*. Crichton *et al*.^28^, both showed a rapid decrease in normalised force to approximately 0.7 of the original force, then a slow decrease towards ~0.4 at 10 s and did not appear to be influenced by tip contact area. Furthermore, Crichton *et al*. showed that the VE is the most viscoelastic of all three skin layers^33^. Jee and Komvopoulos suggested time-dependent deformation of skin is mainly attributed by the cellular epidermis and dermis layers^49^. From our study, we suggest that the VE is likely the main contributor of viscoelasticity in full-thickness skin, as a more prominent viscoelastic behaviour is related to higher VE proportion in the skin composition (Table 2). An example would be the VE in full thickness skin of mice and rats were higher at ~4–9% and 6–9% for small and large pigs, whereas for rabbits and humans, this figure was lower at ~2–2.5%.

Furthermore, both the relaxation profiles of small and large pigs resembled each other and appeared closer to mouse and rat skin rather than to humans (as seen in the normalised load after 10 s in Fig. 2(c)). Despite differences in mass, we found relaxation profiles of rabbit skin behaved similar to human skin. We interpret these as likely due to fluid within the living layers of the skin i.e. intercellular space, and to a lesser extent the presence of collagen within the dermis, also known to behave viscoelastically^50^. Age could be another factor, as while the VE of smaller pigs was lower than larger pigs, the relaxation curves appeared similar – younger skin may be more viscoelastic. Interestingly, Prony coefficients did not appear to vary much between species (0.65–0.78), suggesting allometric scaling trends between species may not be prominent.

On elastic modulus, we anticipated the skin’s elastic modulus would increase with species mass, however, human skin was one of the most compliant. We propose that variations in skin layer thickness ratios of humans affected this (i.e. greater VE layer influence, which was found to be the most compliant of all three layers^33^), although other factors such as hair follicles, captive or environmental exposure differences and skin sites (to a lesser extent) may be relevant. Skin deformation contributed by the SC primarily was suggested by Jee and Komvopoulos^49^. Indeed, this agreed with Fig. 4(b) that the SC was the main layer for stiffness contribution, and that thicker SC corresponded to higher elastic modulus (e.g. small vs. large pigs). Comparing our data with literature, Crichton *et al*.^28^ reported 860 kPa for mouse skin, using Equation 4, which was the trend fitted to the experimental data, we obtained a slightly lower estimate of 53 kPa. However, another example^37^ reported pig skin reduced modulus of 3.77 MPa (tip radius 20 µm), matching well with our estimate of 2.52 MPa (small pigs) and 5.69 MPa (large pigs). Overall, we have shown that the composition of skin is an important factor in influencing the mechanical properties of full thickness skin. Regarding allometric scaling of elastic modulus to body mass presented in Fig. 3(f), the smallest three tips showed consistent logarithmic increase, while the largest tip exhibited a decrease, although this was most likely due to limitations of testing very large tips on mouse and rat skins (Materials and methods - indentation tips).

In terms of scale effects, we found increasing tip contact area correlated with decreasing tissue elastic moduli shown in Fig. 3(e). This tip to modulus trend plotted as log-log gradients for each species showed similar slopes. Previous studies showing this trend proposed that corneocytes of the SC were distributing loads more effectively at smaller contact areas due to corneocyte-to-tip scales^28,33^. Smaller pigs were found to have lower elastic modulus than larger pigs, despite similar overall skin thickness, suggesting scale effects could be more pronounced with certain skin layers, cell structure at different ages, or simply due to higher biological variation in large pig skin specimens, as shown in Supplementary Fig. S5 (with differences between specimens at approximately 1–1.5 orders of magnitude).

On the analytical model (Results - linear analytical model), it provided fresh insights into the skin’s elastic modulus as a function of animal mass across the species tested. For example, by knowing the elastic modulus of a mouse, we could approximate for humans with the mass. The purpose to develop the model was to help interpret our results; to understand the contribution of tissue layers, rather than to provide accurate solutions to solve for the skin properties (for this approach, alternative methods such as finite element analysis would be employed). As such, this model was purely elastic and did not account for viscoelastic and other effects, but still gave reasonable correlation between species. By normalising the data of tip radius and skin thickness, a clearer relationship between elastic modulus and species could be observed, supporting our hypothesis that the main driver of skin’s elastic modulus is the thickness of the skin layers. Other more complex skin modelling approaches utilise finite element analysis (e.g.^51^) which is time consuming and expensive. For many purposes, this model serves as an engineering tool that can provide estimates of the skin’s mechanical properties. Limitations to this model may include inapplicability of species from harsher habitats or outside the scales herein. For example, an elephant weighing ~4 tons, has a skin thickness of ~17.5 mm^52^, but Equations 5–7 suggests a full skin thickness of approximately half at 9.7 mm. However, other species within the mass range investigated, such as sheep, which typically weighs ~40 kg at adulthood, has a skin thickness of 1.83–2.15 mm^53^. This compares well with the same formulae, which estimated a full skin thickness at ~2.3 mm. Based on this information, if we use our estimated mean SC:VE:D ratio of 1:4:95, using Equations 15–17, we could estimate that indenting using a tip radius of 0.18 mm would give a bulk elastic modulus of sheep skin of 422 kPa, which is comparable to Manan & Mahmud’s study who reported a tensile modulus of 369–539 kPa^54^.

In terms of freshness of skin and its mechanics, we endeavoured to work with freshly excised skin. However, *ex vivo* human skin samples obtained were not able to be tested immediately after surgical excision and were refrigerated for up to 24 hours before indentation. Some studies show differences in tissue mechanical properties after storage in refrigerated conditions^55^, and changes to Young’s modulus after storage in freezing conditions^27^. However, studies such as Banga showed minimal changes to tissue quality stored under refrigeration that were used within a few days after excision^56^. Regardless, we did not observe any visual degradation to sample quality between obtaining the skin and completing the experiment, and that current protocols obtaining and processing skin are unlikely to have influenced tissue mechanics. Further, to demonstrate data applicability to *in vivo* human conditions, we compared *ex vivo* skin with *in vivo* conditions with volunteers. In force-relaxation experiments, *in vivo* human skin showed less decrease in force over the initial 2–3 seconds vs. *ex vivo* conditions. We hypothesised additional pressure from blood circulation and skin in its original tension and state could contribute to this difference. Previous porcine studies on brain tissue also showed discrepancies in material stiffness between tissue states^57,58^. This is particularly relevant where development of new devices is performed on excised tissue, with the expectation that translation to live humans will be simple. Despite this, we propose they are unlikely to affect most tests of micro-scale devices, as the differences are small; *ex vivo* skin could still provide valuable data when emulating *in vivo* conditions for certain purposes, such as mechanical testing.

Mechanical testing of medical devices often commences on small animal models during pre-clinical stages. Our data can help enable rapid translation from laboratory conditions by streamlining and reducing animal usage before human testing with improvements being made to devices more effectively. Based on date presented here, we highlight that *ex vivo* human skin, in combination with rabbit and/or small pig skins may be suitable for testing micro-medical devices in developmental stages to obtain a translatable elastic and viscoelastic result. Indeed, the ability to relate animal model experiments to human clinical applications is applicable to several medical interventions, such as in surgery and soft tissue cutting, impact and puncture of skin, and skin based diagnostic and sensing devices, which can be optimised to target the skin at a more precise and effective level.

## Conclusions

To address the need for effective and rapid translation of micro-scale medical devices from laboratory to clinics, we investigated the morphology and the mechanical properties of the skin of mice, rats, rabbits, pigs and humans; with histology and indentation. We hypothesised that we could identify a relationship between these species for elastic modulus and skin thickness using a simple allometric correlation to species mass. We reported this relationship using experimental trends, which correlated well with reported literature. Our data suggest that the thickness of each layer of skin increased with species mass, together with the elastic modulus (except for human skin). We extended this to humans and observed a reduction in material stiffness from *ex vivo* skin to *in vivo* skin. Using indentation, our measured elastic moduli of the selected species is between 10^2^–10^6^ Pa with indenter tip radii between 0.18–3.15 mm. From our study, we recommend that *ex vivo* human skin, rabbit skin and small pig skin would be suitable for pre-clinical testing of medical devices. By establishing the relationships between animal models and humans, we can help translate devices more rapidly through the knowledge of understanding the mechanical properties of the skin and its reaction to a micro-scale like device acting on its surface.

## Materials and Methods

In this paper, we used indentation to measure elastic and viscoelastic properties of skin specimens from mice, rats, rabbits, pigs and humans. We then fitted the Ogden hyperelastic model and two-term Prony series to the loading and force-relaxation curves, to obtain these properties respectively.

### Skin tissue preparation for indentation

Skin was collected from five species: mouse (flank), rat (flank), rabbit (flank), pig (ear) and human (abdomen) spanning four orders of magnitude in mass: mouse ~30 g, rat ~300 g, rabbit ~3 kg, pig ~30 kg, and humans at ~70 kg. In addition, larger, abattoir sourced pigs ~130–150 kg were compared (i.e. from animal experiments and commercial abattoir). This provided comparison between the same species but at two body masses. The mass of the first four species were measured directly from the animals (large pigs were quoted directly from the abattoir), and humans for *ex vivo* skin were estimated from Walpole *et al*.^59^. The mass of the human (*in vivo*) was collected directly from volunteers.

Mice (CD1, female, 10 ± 1 weeks old), rats (Wistar, female, 12 ± 1 weeks old), rabbits (New Zealand white, female, 12 ± 2 weeks old) and small pigs (~20 kg) (Large White, female, 9 ± 1 week old) were obtained from the University of Queensland Biological Resources (St Lucia QLD, Australia). Skin sites selected were from large, uniform areas of the body and avoided weight-bearing regions with thicker SC thicknesses^41^. Flank (mouse, rat, rabbit) or dorsal ear (pig) skin tissue was excised for testing immediately post euthanasia (mouse/rat with CO_2_ chamber, rabbits and pigs with overdose of ketamine/xylazine). Ear skin of large pigs (Large White female, >1 year old) was purchased from Highchester Meats Ltd (Gleneagle QLD, Australia) with skin excised from dorsal ear cartilage without post-cull treatment i.e. hot water dip. Human skin was sourced from the Princess Alexandria Hospital (Herston QLD, Australia) from female abdominoplasty patients aged 36 ± 7.8 years old (mean ± SD). Animal hair was removed with hair clippers (Pet grooming kit, Wahl, Stirling IL, USA) followed by a razor blade shave (Xtreme3, Schick, St Louis MO, USA). Fat was removed from the skin by scalpel. *In vivo* volunteer human skin (dorsal forearm with no visible scarring or defects) was also compared against *ex vivo* human skin (healthy 3 males and 2 females, 24 ± 1.5 years old, mean body mass 63 ± 7.6 kg).

Mechanical testing of skin was completed within three hours post euthanasia, except for pig and *ex vivo* human skin, where supplies were not available on demand – testing was completed within 48 hours of obtaining skin samples. In this situation, skin samples were excised with hydration and viability maintained, similar to Jee and Komvopoulos^37^, except placing the bottom side of skin on cell culture media (RPMI 1640 Medium, Gibco, Thermo Fisher Scientific, Waltham MA, USA) (not submerged) with antibiotics (Ampicillin, Gibco, Thermo Fisher Scientific, Waltham MA, USA) refrigerated at 4 °C. It was ensured the surface was dry to avoid potential changes to the epidermal mechanical properties^29,60^. Skin was returned to room temperature before testing.

All animal work carried out has been approved by the University of Queensland Animal Ethics Committee (ethics number ANRFA/AIBN/473/15). All human work carried out has been approved by the University of Queensland Human Research Ethics Committee (ethics numbers 2008001342 and 2017000693). Written informed consent was obtained from all participants. All experiments were carried out in accordance with the University of Queensland guidelines and regulations.

### Histology

Five individual skin samples of each species were collected for skin thickness measurement. Frozen-section method was selected over paraffin due to less exposure to processing and faster turnaround time. Subcutaneous layer was removed during the dissection. Skin was cut to ~1 cm^2^ size and submerged in 10% neutral buffered formalin (NBF) (HT501128, Sigma Aldrich, St Louis MI, USA) following standard histology protocol^59^ immediately after harvest. Samples were embedded in moulds (Peel-A-Way, Polysciences, Warrington PA, USA) with sectioning matrix (Tissue-Tek OCT, Sakura Finetek, Alphen aan den Rijn, the Netherlands) and frozen by liquid nitrogen. Samples were pinned during fixation and held up right during freezing to ensure perpendicular sections were obtained at 14 µm thick (Microm HM 560, Thermo Fisher Scientific, Waltham MA, USA) and at least three slides were collected from each specimen (subject to quality of sections obtained). Between each slide (Superfrost Plus, Thermo Fisher Scientific, Waltham MA, USA), at least 350 µm of specimen was discarded so sections collected were not adjacent to each other.

Sections were imaged with confocal microscopy (LSM 510 META, Zeiss, Oberkochen, Germany) using 10x and 20x objectives and plain, white light to observe morphology and skin layers. An 800 nm laser was used to identify the presence of collagen at 430 nm, which indicates the approximate dermal layer^62^, if layers were difficult to distinguish. Representative images are shown in Supplementary Fig. S7. Five replicates of each species were measured at least 20 times, up to 100 times on each skin layer depending on species and sample quality (i.e. no folding, curling and/or shattering of histology specimens) (Zen Black Edition 2009, Zeiss, Oberkochen, Germany). Distance was taken as perpendicular to the SC surface and spaced approximately three times the SC length apart between each measurement illustrated in Supplementary Fig. S8. Bright-field microscopy with haematoxylin and eosin following staining^61^ was also used to identify the separate skin strata (BX45, Olympus Corporation, Tokyo, Japan), at 4x, 10x and 40x magnification. Brightness, contrast and colour balance were adjusted (Photoshop CC, Adobe Systems Incorporated, San Jose CA, USA).

### Indentation equipment

Species were separated into thin and thick skin. For thin skin (mice and rats), a Triboindenter (Hysitron TI900, Minneapolis MN, USA) with a MultiRange NanoProbe transducer was used. For the remaining, thick skin, a universal testing machine (Instron 5543, Norwood MA, USA) with a 5 N load cell were used. Both equipment has an overlapping indentation rate of 100 µm s^−1^. The reason for the separation is due to Triboindenter’s maximum vertical displacement of ~90 µm, not deep enough for thicker skin. Secondly, for small displacements (e.g. up to 50 µm), Instron data contained relatively high levels of noise. For these indentations, the Triboindenter was used.

### Polydimethylsiloxane (PDMS) as a handling layer for indentation

A PDMS layer was used as handling layer for the skin to be pinned on, and to protect the load cell in case of overshooting the intended displacement. PDMS backing was made using Sylgard 184 Silicone Elastomer Kit (Dow Corning, Midland MI, USA) mixed with the supplied curing agent in a 20:1 ratio. Vacuum chamber removed air bubbles in mixture. Mixture was poured in circular mould to 7–10 mm thick and cured in 60 °C oven for two hours.

### Indentation tips

Custom-made, aluminium, flat cylindrical tips with radii of 0.180, 0.315, 1.000 and 3.150 mm were used. These radius sizes gave contact area one order of magnitude larger than the previous (except the 0.18 mm tip, which was the smallest we manufactured) to provide a range of readings and extrapolation of material properties outside the scales tested down to the sub-cellular scale^28^. Tip sizes were selected based on Wayes *et al*., who indicated a suitable range of indentation tip size between 0 and 100% of the specimen thickness, as tip sizes significantly larger than the skin thickness changes the experiment to a flat plate compression model^63^. Smaller tip sizes in the micrometre range are more closely associated to and more relevant to the typical scales of microneedle devices.

### Surface roughness

The gap between skin and tissue due to surface roughness may affect mechanical analysis, however, to minimise this effect, skin furrow and hair root regions were avoided. Skin roughness surface amplitudes shown in literature were smaller than our indentation depths (e.g. mice *R*_*a*_ (arithmetic mean) ~7.8^64^, human *R*_*µ*_ (root mean) ~22–30^65^).

### Indentation procedure

A diagram illustrating the method is shown in Supplementary Fig. S9. Skin was placed on wet paper towel moistened with 1x phosphate buffered saline (PBS) during experiment to prevent dehydration^37^. Mice and rat skin (with 1x PBS paper towel layer) were placed on a Triboindenter specimen holder. Rabbit, pig and human skin were pinned on the edges together with 1x PBS paper towel on a PDMS handling layer using hypodermic needles (to its original dimensions prior to excision to mimic *in vivo* conditions) for the Instron stage. Indentation area excluded regions near sample boundaries and the pins. Mice and rat skin were not pinned due to loose-skin nature of animals and limited working space in the Triboindenter. A mass-balance experiment (Supplementary Table S9) was carried out to verify skin was not overhydrated through passive capillary diffusion or osmosis. For the *in vivo* human skin experiment, the volunteers rested their arms on the Intron stage.

Indentation depth was set at approximately 10% of the material thickness^66,67^ to eliminate potential substrate effects while maintaining the ability to measure the effects of the full skin thickness. Tests performed using the Instron incorporated a preload of ~1 mN to ensure full contact of the tip and skin surfaces prior to commencing the loading ramp, similar to the automatic contact detection of the Triboindenter. Loading ramp was 0.01 mm s^−1^ and repeated at 0.1 mm s^−1^. This was not pre-conditioning the material, and the magnitude of the pre-load was the minimum readout from the load cell without ambient fluctuations. This was followed by a fixed-displacement hold for 10 s and an unload ramp at the same rate. We doubled the recording hold time (i.e. data points) for *in vivo* human skin to allow better curve-fit due to small body movements detected during measurement (Fig. 3(b)). Increasing the sampling rate allowed a reduction in recording/relaxation duration. Quality of fit (*R* ^2^) was also used as an indicator to determine the minimum required duration without reaching a fully-relaxed plateau. to determine Prony coefficients; force-relaxation could be as short as less than one second^68^. Each condition was repeated five times for each tip size and indentation rate, with five replicates per species to ensure robustness of data allowing for natural variations in biological specimens.

### Data analysis

Derivations to the Prony series and Ogden curve fits are from Crichton *et al*. and Lin *et al*.^33,69^ Force, displacement and time data were obtained from indentation. Two-term Prony series curve was fitted to force-time data of the hold section during indentation as per Wu *et al*.^70^, with the Prony series in general form (Equation 1) and in two-term form (Equation 2):

1 $$G(t)=1-\sum _{i=1}^{N}{g}_{i}(1-{e}^{-\frac{t}{{\tau }_{i}}})$$

2 $$G(t)=1-{g}_{1}(1-{e}^{-\frac{t}{{\tau }_{1}}})-{g}_{2}(1-{e}^{-\frac{t}{{\tau }_{2}}})$$

in which ${g}_{1}$, ${g}_{2}$ are relaxation magnitudes, ${\tau }_{1}$, ${\tau }_{2}$ are time constants, $t=\frac{{x}_{max}}{v}$, where ${x}_{max}$ is the maximum indentation displacement and $v$ is the indentation velocity. Equation 2 gives a value between 0 and 1, which is used to obtain the reduced elastic modulus by multiplying the instantaneous elastic modulus with $G(t)$. Replicates that did not converge for the code to filter ambient vibrations were discarded.

The Ogden hyperelastic model in Equation 3 was used to fit the force-displacement curves previously demonstrated by Lin *et al*.^69^ beyond small-strain definition, applicable for non-linear stress-strain behaviour of soft material indentations (although not dramatic, our range we indent to approximately 10% of the skin thickness). The authors also considered the Ogden model as the most appropriate for biological tissues^71^. Specifically, we fitted the model to the loading curve instead of the unloading curve for conventional indentation^66^, effectively characterising the material properties at the instance the skin is being loaded, in the same manner as a micro-scale medical device being applied onto the skin. Replicates unable to be fitted despite changing the “initial guess” parameters (i.e. *E* and *α*) and boundary conditions were discarded.

3 $$P=\frac{40{E}_{0}{a}^{2}}{9\alpha (1-{\nu }^{2})}[{(1-{\varepsilon }^{\ast })}^{-\frac{\alpha }{2}-1}-{(1-{\varepsilon }^{\ast })}^{\alpha -1}]$$

where $P$ is the load, ${E}_{0}$ is the elastic modulus, $a$ is the tip contact radius, $\alpha$ is the fitting parameter, $\nu$ is the Poisson’s ratio and $\varepsilon$ is the strain (instantaneous indentation depth/skin thickness) for large deformations applicable to this study^69^.

Matlab 2015a and 2016a (MathWorks, Natick MA) were used to automate data processing. Ogden and Prony curves were fit using the nlinfit function. Up to 10% of the initial force-displacement data was excluded from the origin to avoid fitting over noise/movement (*in vivo*) with relatively low forces, ambient noise and transient artefacts.

### Statistical analysis and graph plotting

Skin thickness power law equations were determined using the Matlab (2016a, MathWorks, Natick MA) Curve Fitting Tool. Prism (GraphPad Inc., La Jolla CA) was as follows: (a) Plotting of all graphs. (b) Statistical significance in indentation and thickness data between species using ordinary one-way ANOVA multiple comparisons (Tukey’s multiple comparisons test). Statistical significance levels shown in figures and tables are: ns (P > 0.05), *(P ≤ 0.05), **(P ≤ 0.01), ***(P ≤ 0.001), ****(P ≤ 0.0001). Standard deviation is stated unless otherwise specified. (c) Elastic modulus power law equations were determined using the Nonlinear Regression (log-log line) Tool.

Power curves were fitted to the central trend of each species to obtain an allometric scaling relationship for skin thickness, elastic modulus and indenter tip radius from Tables 3 and 5:

4 $$y=a{x}^{b}\,$$

5 $$\mathrm{log}\,y=\,\mathrm{log}\,a+b\,\mathrm{log}\,x$$

in both power law and logarithmic forms, with the latter resembling the linear algebraic equation $y=mx+c$ for the log-log graphs shown in Figs 1(f) and 3(e,f).

### Analytical model

To investigate whether elasticity can be primarily defined as a function of skin layer thicknesses, the skin was modelled as three balanced, ideal springs in series, with no mass, damping or viscoelastic effects representing each of the skin layer and to isolate the system to the elastic components. Viscoelasticity could be applied later using the Prony coefficients as they do not appear to depend on scale^33^; including damping elements in the model but this complicates the calculation and deviates the model away from its intended purpose. The three spring system was comparable to the work of Pailler-Mattei *et al*., who quantified their tissue layers as dermis, hypodermis and muscle for their indentation study^26^. Starting from a basic spring force-displacement relationship with Hooke’s law:

6 $$F=kx$$

A constant contact area throughout the skin equal to the tip contact area was assumed, i.e. 1D treatment of compression in this model. This was expressed as the material stiffness of an infinite series of springs in series fixed on one end^72^:

7 $$k=[\frac{1}{{\sum }_{i=1}^{n}\frac{1}{{k}_{i}}}]$$

For a three-layer composite structure model defined as the SC, VE and D layers, this becomes:

8 $$F=[\frac{1}{\frac{1}{{k}_{SC}}+\frac{1}{{k}_{VE}}+\frac{1}{{k}_{D}}}]x$$

where $x$ is the displacement of the tip and ${k}_{layer}$ is the stiffness of each layer as the axial stiffness in relation to elasticity:

9 $${k}_{layer}=\frac{{E}_{layer}A}{{t}_{layer}}$$

With $A$ approximated as the tip surface area and ${t}_{layer}$ as the measured thickness of the skin layer and ${E}_{layer}$ as the elastic moduli of the SC, VE and D of mice skin obtained from mice by Crichton *et al*.^33^ fitted using power law (parameters in Supplementary Table S8). Mouse skin layer data was used here, as we hypothesised that skin of different species share common material properties.

A generalised equation to estimate the elastic modulus, if the skin layer thicknesses were known, was developed from the definition of stress, assuming an elastic relationship:

10 $$E=\frac{\sigma }{\varepsilon }$$

11 $$\sigma =\frac{F}{A}$$

Substituting Equations 8–11, we obtained the generalised analytical equation to estimate elastic modulus of skin:

12 $$E=\frac{1}{\frac{1}{{k}_{SC}}+\frac{1}{{k}_{VE}}+\frac{1}{{k}_{D}}}\frac{x}{A\varepsilon }$$

where $x$ is the maximum indentation displacement, $A=\pi {R}^{2}$ for a flat, cylindrical tip and $\varepsilon$ is the maximum strain. The area modifier allowed for tip interface scale dependencies as observed in biological tissues.

A schematic diagram of the simplified skin model is shown in Supplementary Fig. S11.

## Data availability

The datasets generated during and/or analysed during the current study are available from the corresponding author on reasonable request.

## Electronic supplementary material

Supplementary information
