# Supplementary material for: Allometric scaling of skin thickness, elasticity, viscoelasticity to mass for micro-medical device translation: from mice, rats, rabbits, pigs to humans
Source: Sci Rep. 2017 Nov 21;7:15885. doi: 10.1038/s41598-017-15830-7 (PMC5698453; doi:10.1038/s41598-017-15830-7)
Supplement: Supplementary file 1 — Supplementary information [file 41598_2017_15830_MOESM1_ESM.pdf]

Allometric scaling of skin thickness, elasticity, viscoelasticity to mass for micro-medical device translation: from mice, rats, rabbits, pigs to humans

Jonathan CJ Wei[1], Grant A Edwards[2], Darren J Martin[2], Han Huang[3], Michael L Crichton[1,4] and Mark AF Kendall[1,5,6]***

*m.crichton@hw.ac.uk, **m.kendall@uq.edu.au

Supplementary information

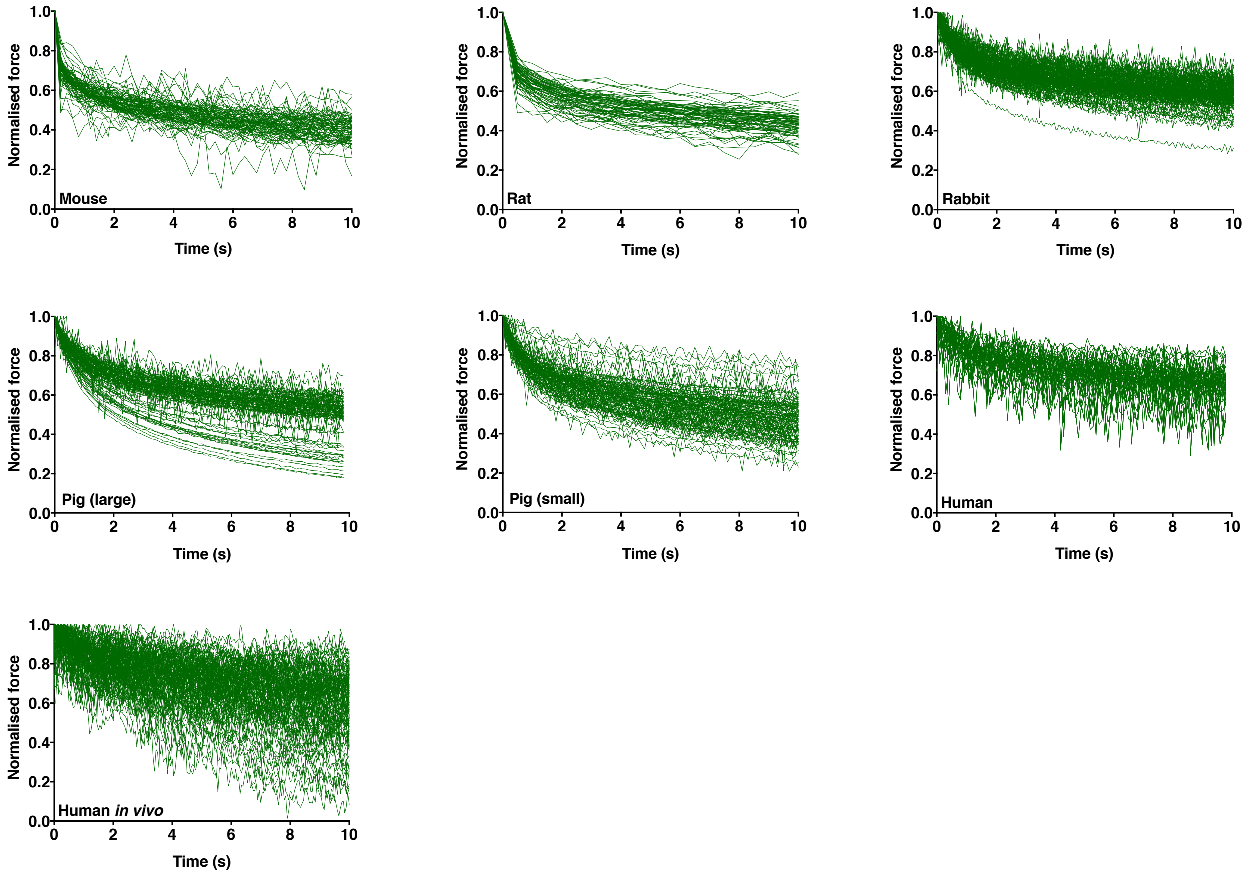

Supplementary Fig. S 1. Raw data of force-relaxation experiment for all species showing relative noise and spread of every replicate. Data presented here has been downsampled for illustration and file size optimisation purposes only. Original data was used for the analysis.

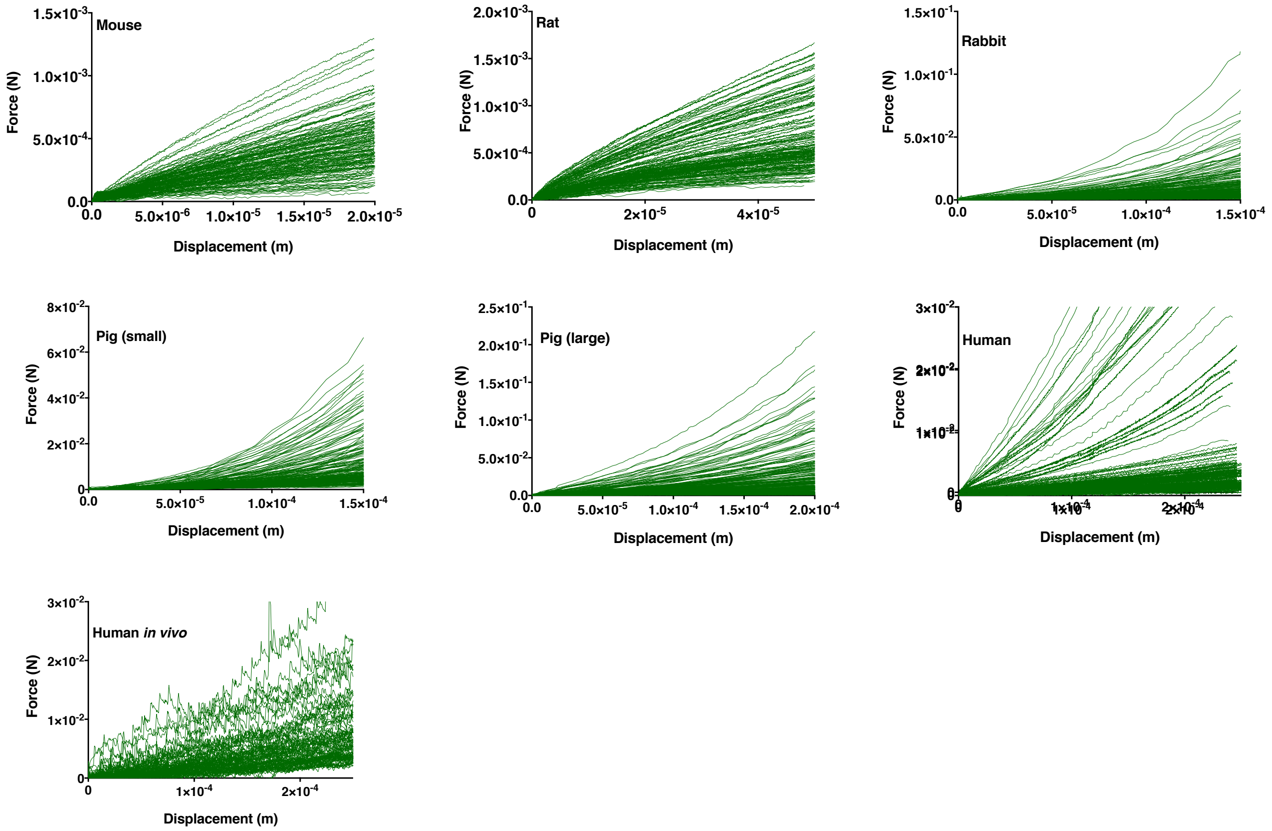

Supplementary Fig. S 2. Raw data of force-displacement curves for all species showing relative noise and spread of every replicate. Note that the y-axes are different between each species. Data presented here has been downsampled for illustration and file size optimisation purposes only. Original data was used for the analysis.

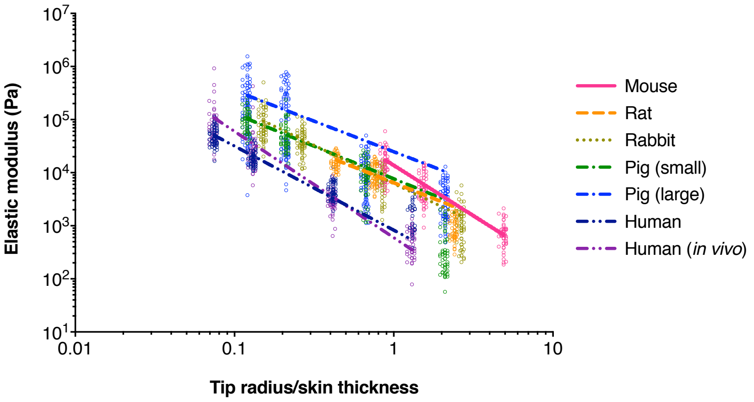

Supplementary Fig. S 3. Elastic moduli shown against indentation tip radii normalised with skin thickness.

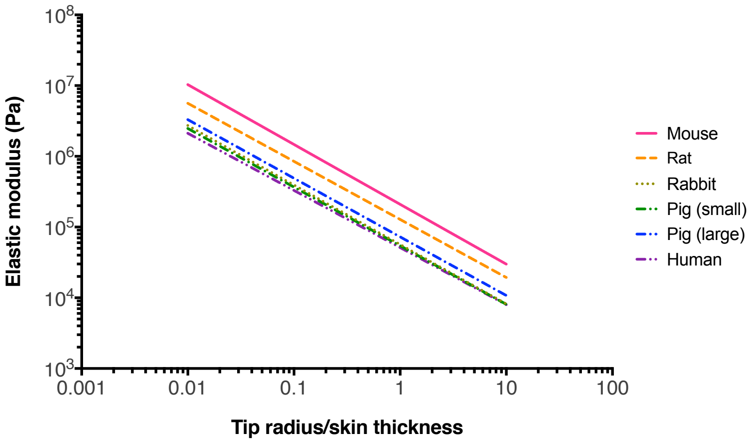

Supplementary Fig. S 4. Analytical model estimation of the elastic moduli against tip radii normalised with skin thickness.

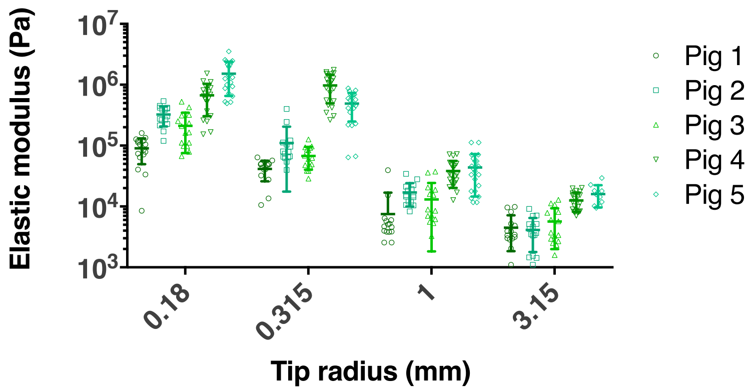

Supplementary Fig. S 5. Elastic moduli of individual pig samples showing the differences between pigs 1-3 and 4-5, where samples 4-5 appeared to have higher *E* than 1-3.

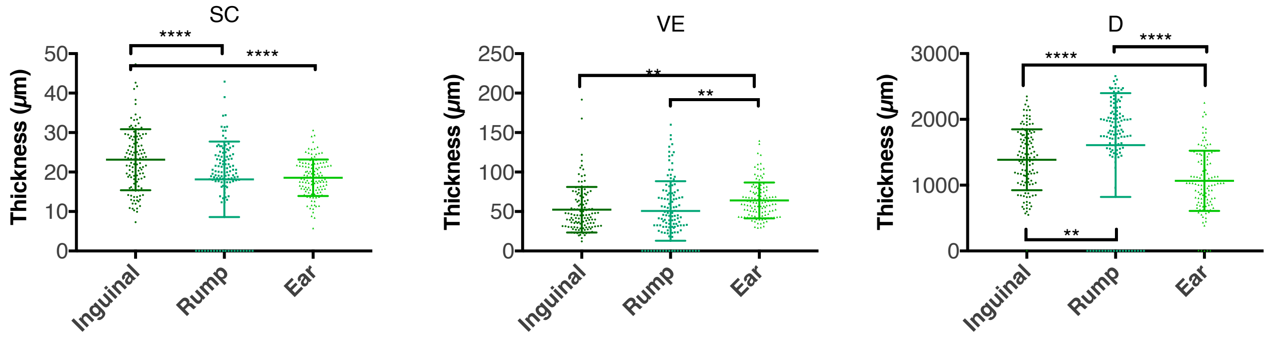

Supplementary Fig. S 6. Comparison between pig inguinal, rump and ear skin layer thickness.

(a) Mouse

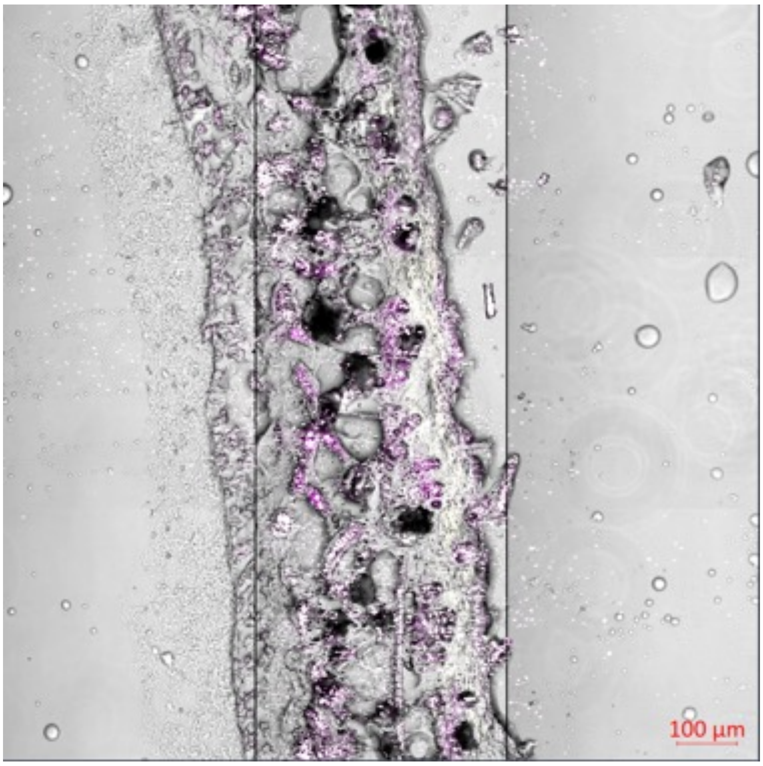

(b) Rat

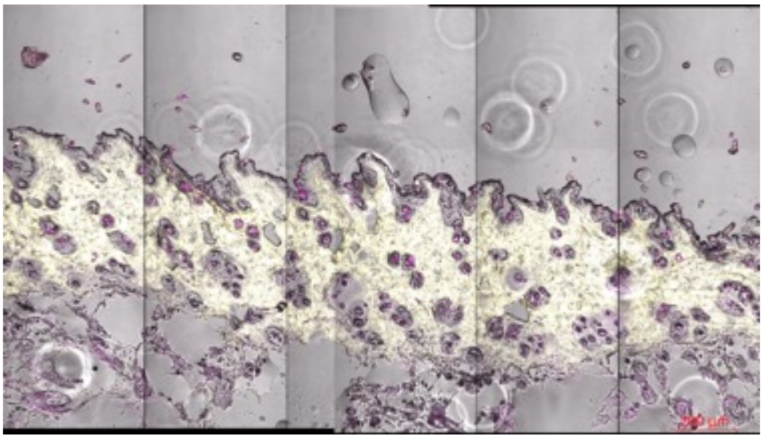

(c) Rabbit

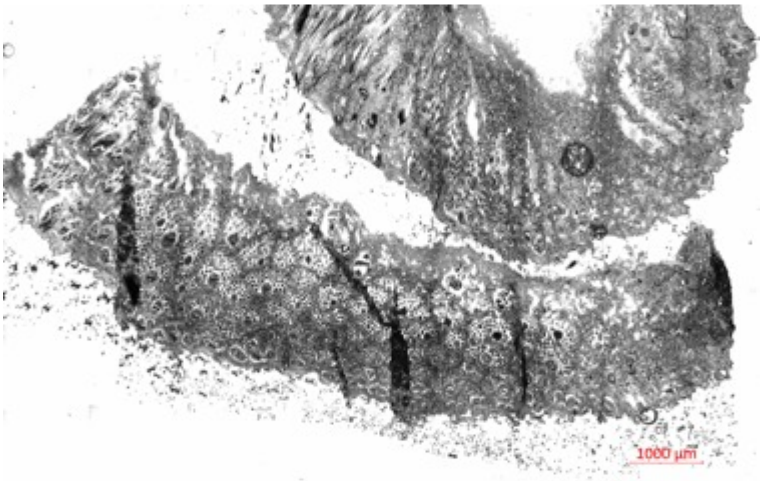

(d) Pig (small)

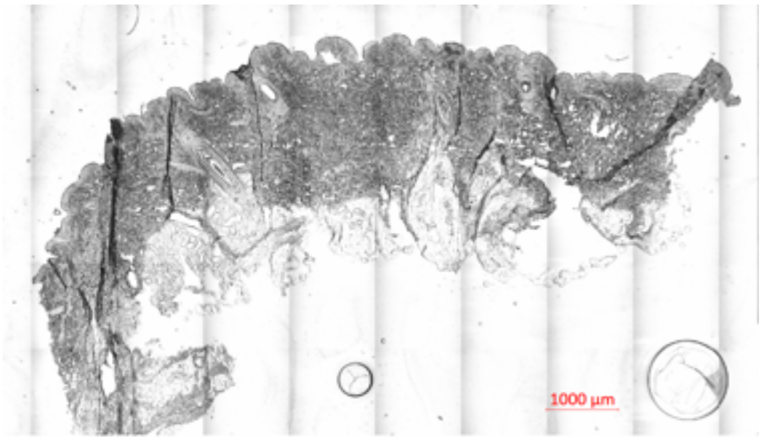

(e) Pig (large)

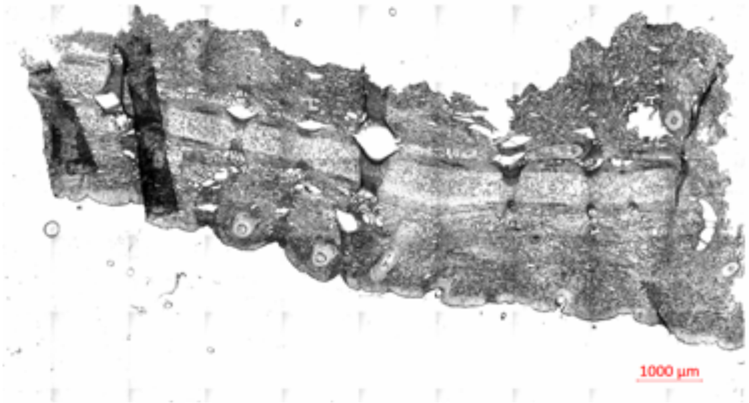

(f) Human

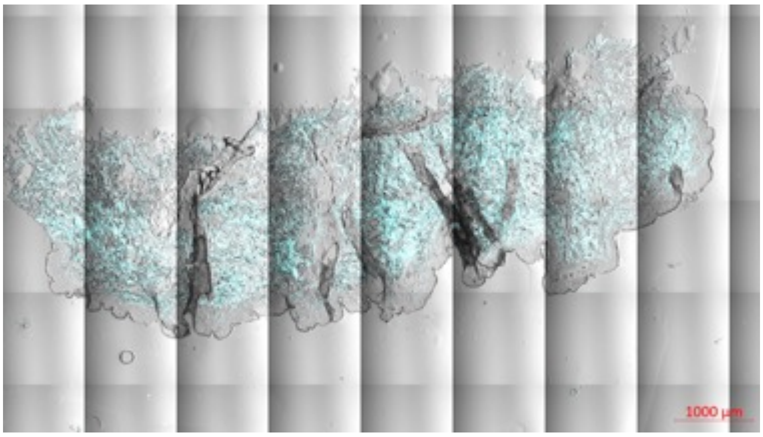

Supplementary Fig. S 7. Representative images captured using the multiphoton microscope used for skin thickness measurements.

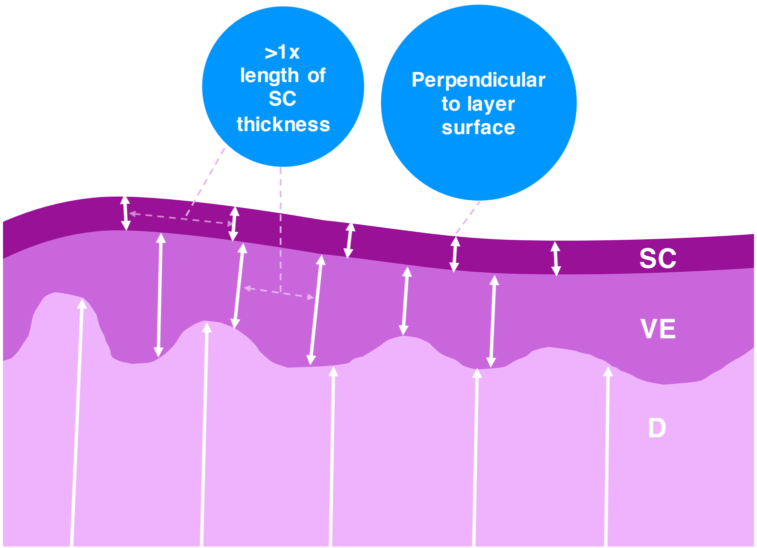

Supplementary Fig. S 8. Illustration of how sections in Figure 1 were typically measured.

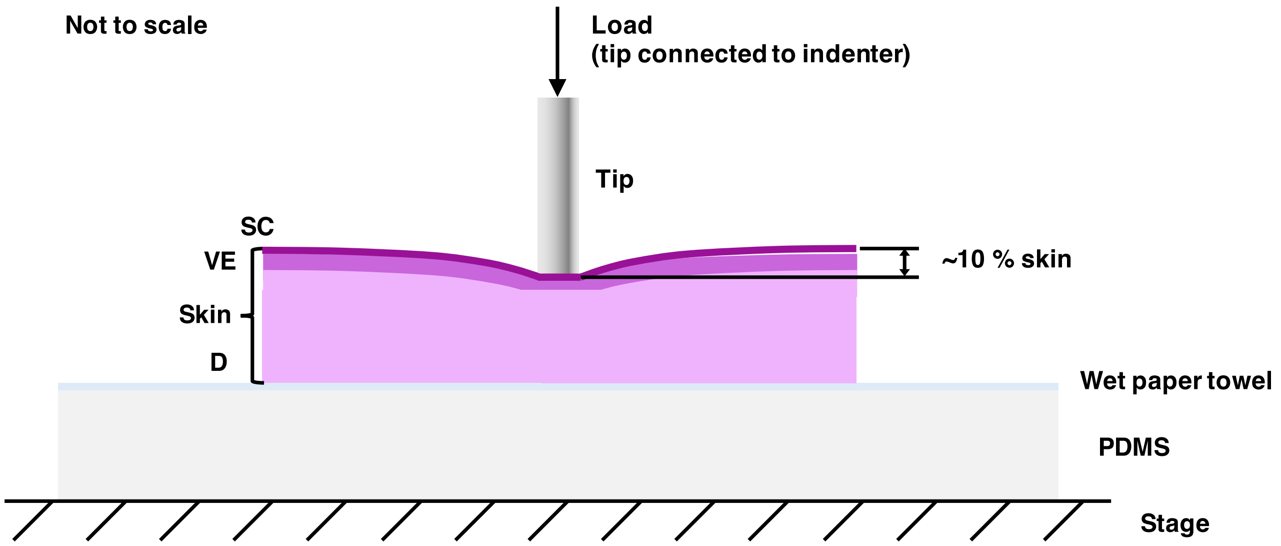

Supplementary Fig. S 9. Schematic diagram illustrating a simplified skin model setup for indentation tests. PDMS or aluminium backings are used as a stage for the indenter.

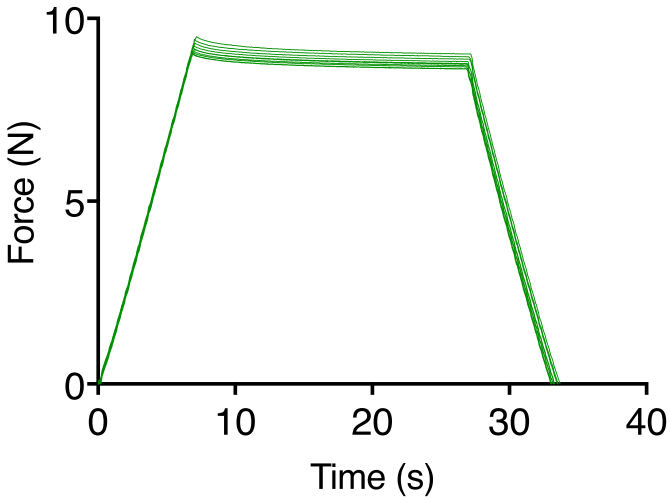

Supplementary Fig. S 10. PDMS raw force-time curves PDMS indentation using the 3.15 mm tip at v = 0.1 mm s-1.

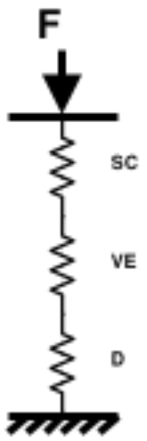

Supplementary Fig. S 11. Illustration of the simplified skin model as a bottom-fixed three springs in series with a force being applied from the tip.

Supplementary Table S 1. Measured mass of species.

Species	*m* (kg)	SD	*Age*	SD/range
**Mouse**	0.0204	± 0.002	10	± 1 week
**Rat**	0.398	± 0.02	12	± 1 week
**Rabbit**	3.50	± 0.5	12	± 2 weeks
**Pig (small)**	20.6	± 1.5	9	± 1 week
**Pig (large)**	140	± 10*	>1	year*
**Human**	n/a	**	36	± 7.8 years
**Human (*in vivo*)**	63	± 7.6	24	± 1.5 years

*approximation quoted from facility manager

**information not provided, estimation used

Supplementary Table S 2. P-values of comparing the skin layer thicknesses between species from **Table 2**. One-way ANOVA Tukey's multiple comparisons test.

**Stratum corneum**	**Adjusted P-values**					
**Species**	**Mouse**	**Rat**	**Rabbit**	**Pig (small)**	**Pig (large)**	**Human**
**Mouse**		****	****	****	****	****
**Rat**	<0.0001		****	****	****	****
**Rabbit**	<0.0001	<0.0001		****	****	****
**Pig (small)**	<0.0001	<0.0001	<0.0001		****	ns
**Pig (large)**	<0.0001	<0.0001	<0.0001	<0.0001		****
**Human**	<0.0001	<0.0001	<0.0001	>0.9999	<0.0001	
**Viable epidermis**	**Adjusted P-values**					
**Species**	**Mouse**	**Rat**	**Rabbit**	**Pig (small)**	**Pig (large)**	**Human**
**Mouse**		ns	****	****	****	****
**Rat**	0.3721		****	****	****	****
**Rabbit**	<0.0001	<0.0001		ns	****	****
**Pig (small)**	<0.0001	<0.0001	0.6224		****	***
**Pig (large)**	<0.0001	<0.0001	<0.0001	<0.0001		****
**Human**	<0.0001	<0.0001	<0.0001	0.0005	<0.0001	
**Dermis**	**Adjusted P-values**					
**Species**	**Mouse**	**Rat**	**Rabbit**	**Pig (small)**	**Pig (large)**	**Human**
**Mouse**		*	****	****	****	****
**Rat**	0.0254		****	****	****	****
**Rabbit**	<0.0001	<0.0001		****	***	****
**Pig (small)**	<0.0001	<0.0001	<0.0001		ns	****
**Pig (large)**	<0.0001	<0.0001	0.0003	0.6806		****
**Human**	<0.0001	<0.0001	<0.0001	<0.0001	<0.0001	

Supplementary Table S 3. Mean Prony series goodness of fit to raw data. The R^2 values appears to correlate with the material stiffness of the skin as the lower the load, the higher the relative ambient noise. Note that Mouse and Rat skins are tested using the Triboindenter and not the Instron.

Species	R^2	SD
**Mouse**	0.927	± 0.135
**Rat**	0.967	± 0.036
**Rabbit**	0.893	± 0.152
**Pig (small)**	0.944	± 0.144
**Pig (large)**	0.958	± 0.079
**Human**	0.646	± 0.264
**Human (*in vivo*)**	0.575	± 0.276

Supplementary Table S 4. Mean Prony coefficient at 100 mm s^{-1}.

Species	*g(t)*	SD
**Mouse**	0.763	0.112
**Rat**	0.657	0.046
**Rabbit**	0.708	0.063
**Pig (small)**	0.681	0.053
**Pig (large)**	0.665	0.073
**Human**	0.784	0.054
**Human (*in vivo*)**	0.680	0.272

Supplementary Table S 5. Mean Ogden curve goodness of fit to raw data.

Species	R^2	SD
**Mouse**	0.939	± 0.115
**Rat**	0.926	± 0.072
**Rabbit**	0.980	± 0.031
**Pig (small)**	0.987	± 0.017
**Pig (large)**	0.980	± 0.046
**Human**	0.932	± 0.067
**Human (*in vivo*)**	0.913	± 0.108

Supplementary Table S 6. Mean Ogden α coefficient for all species.

Species	α	SD
**Mouse**	10.540	± 2.169
**Rat**	10.498	± 2.676
**Rabbit**	27.244	± 12.801
**Pig (small)**	42.139	± 13.927
**Pig (large)**	27.478	± 9.857
**Human**	23.655	± 8.622
**Human (*in vivo*)**	11.446	± 8.982

Supplementary Table S 7. Modelled structural stiffness of each layer and species using **Equation 9**.

		**0.18**	**0.315**	**1**	**3.15**
			***k* (kN m^{-1})**		
	**SC**	2.410	5.230	25.900	127.000
**Mouse**	**VE**	0.144	0.265	0.942	3.320
	**D**	0.817	1.900	10.900	61.900
	**SC**	2.640	5.720	28.400	139.000
**Rat**	**VE**	0.137	0.253	0.898	3.160
	**D**	0.355	0.827	4.750	26.900
	**SC**	0.721	1.570	7.760	38.100
**Rabbit**	**VE**	0.068	0.126	0.446	1.570
	**D**	0.084	0.195	1.120	6.330
	**SC**	0.469	1.020	5.060	24.800
**Pig (small)**	**VE**	0.042	0.078	0.278	0.979
	**D**	0.077	0.179	1.030	5.820
	**SC**	0.469	1.020	5.060	24.800
**Pig (large)**	**VE**	0.042	0.078	0.278	0.979
	**D**	0.077	0.179	1.030	5.820
	**SC**	0.469	1.020	5.060	24.800
**Human**	**VE**	0.042	0.078	0.278	0.979
	**D**	0.077	0.179	1.030	5.820

Supplementary Table S 8. Power law parameters from Crichton et al.[33] fitted using Microsoft Excel (2016 for Mac, Redmond WA).

Skin layer	*a*	*b*
**Stratum corneum**	497.85	-0.614
**Viable epidermis**	10.260	-0.903
**Dermis**	21743	-0.488

Supplementary Table S 9. Mass-balance experiment showing mean mass change of skin during experimentation. Four pieces of skin (rat/pig) were placed in petri dishes with the dermis side exposed to 1x PBS. Excess liquid was allowed to drip and absorbed with tissue paper prior to weighing.

	Percentage change in mass (%)			
**Time (hrs)**	**0**	**1**	**2**	**3**
**Pig skin**	0.00	4.02	4.58	7.26
**Rat skin**	0.00	4.91	3.60	5.42
